# LIM-Only Protein 4 (LMO4) and LIM Domain Binding Protein 1 (LDB1) Promote Growth and Metastasis of Human Head and Neck Cancer (LMO4 and LDB1 in Head and Neck Cancer)

**DOI:** 10.1371/journal.pone.0164804

**Published:** 2016-10-25

**Authors:** Elizabeth A. Simonik, Ying Cai, Katherine N. Kimmelshue, Dana M. Brantley-Sieders, Holli A. Loomans, Claudia D. Andl, Grant M. Westlake, Victoria M. Youngblood, Jin Chen, Wendell G. Yarbrough, Brandee T. Brown, Lalitha Nagarajan, Stephen J. Brandt

**Affiliations:** 1 Department of Cancer Biology, Vanderbilt University Medical Center, Nashville, TN, United States of America; 2 Department of Medicine, Vanderbilt University Medical Center, Nashville, TN, United States of America; 3 Department of Pathology, Microbiology, and Immunology, Vanderbilt University Medical Center, Nashville, TN, United States of America; 4 Department of Surgery, Vanderbilt University Medical Center, Nashville, TN, United States of America; 5 Department of Cell & Developmental Biology, Vanderbilt University Medical Center, Nashville, TN, United States of America; 6 Department of Otolaryngology and Barry Baker Laboratory for Head and Neck Oncology, Vanderbilt University School of Medicine, Nashville, TN, United States of America; 7 Vanderbilt-Ingram Cancer Center, Vanderbilt University Medical Center, Nashville, TN, United States of America; 8 VA Tennessee Valley Healthcare System, Nashville, TN, United States of America; 9 Department of Genetics, University of Texas M.D. Anderson Cancer Center, Houston, TX, United States of America; National University Singapore Yong Loo Lin School of Medicine, SINGAPORE

## Abstract

Squamous cell carcinoma of the head and neck (HNSCC) accounts for more than 300,000 deaths worldwide per year as a consequence of tumor cell invasion of adjacent structures or metastasis. LIM-only protein 4 (LMO4) and LIM-domain binding protein 1 (LDB1), two directly interacting transcriptional adaptors that have important roles in normal epithelial cell differentiation, have been associated with increased metastasis, decreased differentiation, and shortened survival in carcinoma of the breast. Here, we implicate two LDB1-binding proteins, single-stranded binding protein 2 (SSBP2) and 3 (SSBP3), in controlling LMO4 and LDB1 protein abundance in HNSCC and in regulating specific tumor cell functions in this disease. First, we found that the relative abundance of LMO4, LDB1, and the two SSBPs correlated very significantly in a panel of human HNSCC cell lines. Second, expression of these proteins in tumor primaries and lymph nodes involved by metastasis were concordant in 3 of 3 sets of tissue. Third, using a Matrigel invasion and organotypic reconstruct assay, CRISPR/Cas9-mediated deletion of *LDB1* in the VU-SCC-1729 cell line, which is highly invasive of basement membrane and cellular monolayers, reduced tumor cell invasiveness and migration, as well as proliferation on tissue culture plastic. Finally, inactivation of the *LDB1* gene in these cells decreased growth and vascularization of xenografted human tumor cells *in vivo*. These data show that LMO4, LDB1, and SSBP2 and/or SSBP3 regulate metastasis, proliferation, and angiogenesis in HNSCC and provide the first evidence that SSBPs control LMO4 and LDB1 protein abundance in a cancer context.

## Introduction

Worldwide, carcinoma of the head and neck is the sixth leading cancer by incidence and accounts for 300,000 deaths per year. The majority are squamous cell carcinomas (HNSCC), originate in the oral cavity, pharynx, or larynx, and spread by local extension or lymphatic metastasis. These cancers can be treated surgically in many patients; however, residual disease or second primaries result frequently in recurrence [1]. While much is known about causation for this malignancy [2–4], the processes regulating tumor cell function, particularly invasiveness and metastatic potential, have not been fully elucidated.

LIM-only protein 4 (LMO4) is a nuclear adapter critical for the assembly of multi-protein transcriptional complexes that regulate malignant epithelial cell proliferation and differentiation [5, 6]. In addition, LMO4 is a cofactor in SNAIL2-mediated epithelial-mesenchymal transition (EMT) [7] and is an important regulator of gene expression in neural crest cells [8, 9] during embryogenesis. This likely explains the failure of neural tube closure and exencephaly in *Lmo4* knockout mice [8].

Dysregulated LMO4 expression is characteristic of a number of epithelial malignancies, including oral cavity carcinoma, and is associated with reduced tumor cell differentiation and increased lymph node metastasis [10, 11]. Of particular interest to us, LMO4 correlated in abundance and colocalized in tissue sections in HNSCC with its interaction partner, LIM domain-binding protein 1 (LDB1) [11]. LMO4 is overexpressed, in addition, in alveolar rhabdomyosarcoma [12] and carcinoma of the breast [5, 6, 13, 14], and all three remaining LIM-only family members, LMO1, LMO2, and LMO3, may also be oncoproteins. LMO1 and LMO2 expression is dysregulated by multiple mechanisms in T-cell acute lymphoblastic leukemia (reviewed in [15, 16]), LMO2 is overexpressed in prostate cancer [17], and LMO3 is upregulated in neuroblastoma [18].

Through studies of LMO2 protein turnover in erythroid cells, we identified a novel function for a small family of LDB1-interacting proteins–protection of LDB1 and its LMO interaction partners from ubiquitylation and proteosomal degradation [19]. Both single-stranded binding protein-2 (SSBP2) and -3 (SSBP3), were shown to competitively inhibit LDB1 ubiquitylation by its E3 ubiquitin ligase, RING finger LIM domain-binding protein (RLIM) [19], and thereby reduce proteasome-mediated turnover. Here, we demonstrate that LMO4 and LDB1 expression correlated closely with that of SSBP2 and SSBP3 in a panel of human oral cavity carcinoma cell lines, that these proteins were concordantly expressed in oral cavity and oropharyngeal tumor primaries and lymph node metastases, and that LDB1 gene inactivation significantly inhibited cellular invasiveness and proliferation and tumor angiogenesis.

## Materials and Methods

### Cell lines and treatments

Human oral cavity carcinoma cell lines SCC-4 (catalog number CRL-1624), SCC-25 (catalog number CRL-1628), SCC-9 (catalog number CRL-1629), SCC-9 (catalog number CRL-1629), SCC-15 (catalog number CRL-1623), and Cal-27 (catalog number CRL-2095) were obtained from ATCC, SCC-61 and UM-SCC-47 were contributed by Dr. Wendell Yarbrough (Vanderbilt University), HN-SCC-131 was provided by Dr. Susanne Gollin (University of Pittsburgh), and VU-SCC-1352 and VU-SCC-1729 came from the Barry Baker Laboratory for Head and Neck Oncology at Vanderbilt University, where they were derived. These lines were unlinked to any clinical information or identifying information. All oral cavity carcinoma cell lines were grown in Dulbecco’s modified Eagle medium (DMEM; Life Technologies-Thermo Fisher Scientific, Grand Island, NY) supplemented with 10% fetal bovine serum (FBS, Atlas Biologicals, Fort Collins, CO) and 1% penicillin/streptomycin and incubated in 5% CO_2_ at 37°C. Fetal esophageal fibroblasts were grown in DMEM supplemented with 10% FBS and 1% penicillin/streptomycin and cultured in 5% CO_2_ at 37°C.

### Antibodies

LDB1, LMO4, SSBP2, and SSBP3 were detected by immunoblot and immunohistochemistry analysis using antibodies that were generated or purchased commercially as follows. Polyclonal antibodies to LDB1 (sc-11198), β-actin (sc-44990), and glyceraldehyde phosphate dehydrogenase (sc-9485) were purchased from Santa Cruz Biotechnologies (Dallas, TX). Rat monoclonal antibody to LMO4 was provided by Dr. Jane Visvader (Walter and Eliza Hall Institute, Melbourne, Australia) and has been described [20]. Affinity-purified polyclonal rabbit antibody to SSBP2 was prepared by contract by SDIX (Newark, DE) using a synthetic polypeptide antigen corresponding to amino acids 163–243. Polyclonal rabbit antibody to SSBP3 was contributed by Dr. Lalitha Nagarajan and has been described previously [21].

### Immunohistochemistry analysis

Oral cavity carcinomas, oropharyngeal carcinomas, and surgically dissected lymph nodes for both were obtained during operative procedures at Vanderbilt University Hospital. Within 30 minutes of removal, tissue was placed in a biopsy cassette and immersed in 10% formalin for 24–48 hours. Cassettes were then moved to 70% ethanol for paraffin embedding and sectioning. Immunohistochemistry analysis was performed in the Translational Pathology Shared Resource (TPSR) at Vanderbilt University as follows. Slides were de-paraffinized and heat-aided antigen retrieval was carried out for 10 min. They were then incubated with a 1:500 dilution of LDB1 antibody, 1:700 dilution of LMO4 antibody, 1:1200 dilution of SSBP2 antibody, or 1:500 dilution of SSBP3 antibody for one hour and the appropriate biotinylated secondary antibody for 30 min. Finally, slides were dehydrated, cleared, and cover-slipped before analysis. Every experiment included sections incubated with secondary antibody alone or an isotype control antibody plus secondary antibody as appropriate.

Immunohistochemistry analysis was carried out on sections of paraffin-embedded tumor from three individuals with carcinoma of the oral cavity. Clinical stage ranged from I-IV and tumor grade from moderately- to well-differentiated. All three were p16^INK4A^-negative, and two had metastasized to cervical lymph nodes. Tumors from 7 individuals with carcinoma of the oropharynx were also analyzed. All expressed p16^INK4A^, a surrogate for HPV positivity [22], and three of the seven had metastasized to cervical lymph nodes. Clinical samples were supplied and exclusively handled by a qualified surgical pathologist and were unlinked to identifying information. All described studies using de-identified human tissue samples and cell lines were exempt from purview by the Institutional Review Board.

### CRISPR/Cas9 mediated mutagenesis of *Ldb1* gene

The CRISPR/Cas9 system was used to target LDB1 essentially as described [23] using the following guide RNAs (gRNAs): LDB1 gRNA1 forward, CACCGACCATGCTGGATAGGGATGT; LDB1 gRNA1 reverse, AAACACATCCCTATCCAGCATGGT; LDB1 gRNA2 forward, CACCGGTAGGCGGATACATGGGAGT; LDB1 gRNA2 reverse, AAACACTCCCATGTATCCGCCTAC (Integrated DNA Technologies, Coralville, IA). VU-SCC-1729 cells were transduced with retrovirus expressing double-stranded RNAs targeting LDB1 exon 1 (gRNA1), exon 2 (gRNA2), or empty vector. Cells were selected with 7 μg/ml puromycin, which was added to medium 36 hr after transfection. Individual colonies were isolated, transferred first to a 96-well plate in selective medium, and then expanded.

### Immunoblot analysis

Immunoblot analysis was carried out as previously described [24] and band intensities quantified using ImageJ software. The abundance of LDB1, LMO4, SSBP2, and SSBP3 was normalized to β-actin and related to that of the highest expresser.

### *In vitro* proliferation assay

VU-SCC-1729 and VU-1729-2:7 cells were incubated in 6-well plates containing DMEM plus 10% FBS and 1% penicillin/streptomycin at 5.0% CO_2_ and 37°C. A total of 46,000 cells/well was inoculated initially into each of four well and the contents of one well was taken daily for viable cell count by trypan blue exclusion. The assay was performed in triplicate. Student’s t-test was used to evaluate the statistical significance of differences in mean cell number. P < 0.5 was considered statistically significant (GraphPad Software, La Jolla, CA).

### Matrigel invasion assay

Invasion was measured using a modified Matrigel method [25]. Briefly, the superior surface of the membrane in a Transwell chamber with 8.0 μm pores (Costar Corning, Corning, NY) was coated with a 1:5 mixture of Matrigel (BD Biosciences, Franklin Lakes, NJ) in Opti-MEM and its inferior surface coated with a 1:20 mixture. After the Matrigel solidified, the chamber’s surfaces were blocked with 1% bovine serum albumin for 30 min at 37°C. VU-SCC-1729 cells and derivatives were cultured for 24 hr in Opti-MEM, after which 100,000 of these serum-starved cells were added to the upper chamber. Chambers were submerged in DMEM containing 10% FBS and 1% penicillin/streptomycin and incubated at 37°C. Cells were fixed with 10% formalin after 6 hr, stained with crystal violet, and counted. Data derived from a minimum of two biological replicates. Student’s t-test was used to determine the significance of differences in mean cell number +/- SEM. P < 0.5 was considered statistically significant (GraphPad Software, La Jolla, CA).

### Organotypic construct assay

Organotypic construct assays with the VU-SCC-1729 and VU-SCC-1729-2:7 cell lines were carried out exactly as described [26]. Cultures were harvested on day 17, fixed in 10% formalin overnight, and paraffin embedded, sectioned, and stained with hematoxylin and eosin in the TPSR. Assays were performed in duplicate. The area of invasion was determined from multiple images of tissue sections and quantified using ImageJ software. Student’s t-test was used to determine the significance of differences in mean cell number +/- SEM. P < 0.5 was considered statistically significant (GraphPad Software, La Jolla, CA).

### Xenograft assay

Athymic male nude mice were obtained from The Jackson Laboratory (Bar Harbor, ME) and housed under pathogen-free conditions. For tumor progression studies, 4–5 week-old nude mice received subcutaneous injections of 500,000 parental or VU-1729-2:7 cells mixed with Matrigel in a volume of 100 uL per mouse as described previously [27]. Parental cells were injected into the left flank and the corresponding CRISPR/Cas9 LDB1 knockout cells into the contralateral flank. Tumor volume was measured using calipers according to the following formula: volume = length × width^2^ × 0.52 [28]. A total of 10 mice were used and their tumors processed for histology 23 days post-injection. Tumor xenografts were dissected, fixed in 10% formalin for 48 hr, and brought to the TPSR for embedding, sectioning, and staining.

Immunohistochemistry analysis for cleaved caspase 3 was also carried out by the TPSR. PCNA and vWF immunofluorescence microscopy were performed as described [27, 29] and photomicrographs taken through an Olympus CK40 inverted microscope (Olympus America, Center Valley, PA) equipped with an Optronics DEI-750C charge-coupled-device camera. CellSens imaging software (Olympus) was used to quantify microvessel density through enumeration of vWF-positive blood vessels. Data derived from analysis of 5–7 tumors per condition, and Student’s t-test was used to determine the significance of differences in mean ± SEM (GraphPad software). P < 0.5 was considered statistically significant.

This work was carried out in accordance with the recommendations of the Guide for the Care and Use of Laboratory Animals of the National Institutes of Health. Mice were used as described with the approval of the Institutional Animal Care and Use Committee of Vanderbilt University (protocol number M/13/092). Implantation of tumor cells was carried out under isoflurane anesthesia and every effort was made to minimize suffering. Carbon dioxide inhalation in a dedicated chamber was employed for euthanasia of mice.

### RNA-seq analysis

Total RNA was isolated from VU-SCC-1729 and CRISPR-Cas9 LDB1 knockout (VU-1729-2:7) cells cultured in triplicate. RNA-seq libraries were generated and sequenced by the Vanderbilt Technologies for Advanced Genomics Core Shared Resource (VANTAGE, Vanderbilt University, Nashville, TN). Bioinformatic analysis was carried out by Dr. Yan Guo (Vanderbilt University) and RNA-seq data also analyzed using iPathwayGuide (Advaita Bio, Plymouth, MI; http://www.advaitabio.com/ipathwayguide). RNA-seq data were submitted to Gene Expression Omnibus and assigned accession number GSE79183 (https://www.ncbi.nlm.nih.gov/geo/query/acc.cgi?acc=GSE79183).

### Results Concordance of LMO4, LDB1, and SSBP expression in human oral cavity carcinoma cells

**Fig 1:**
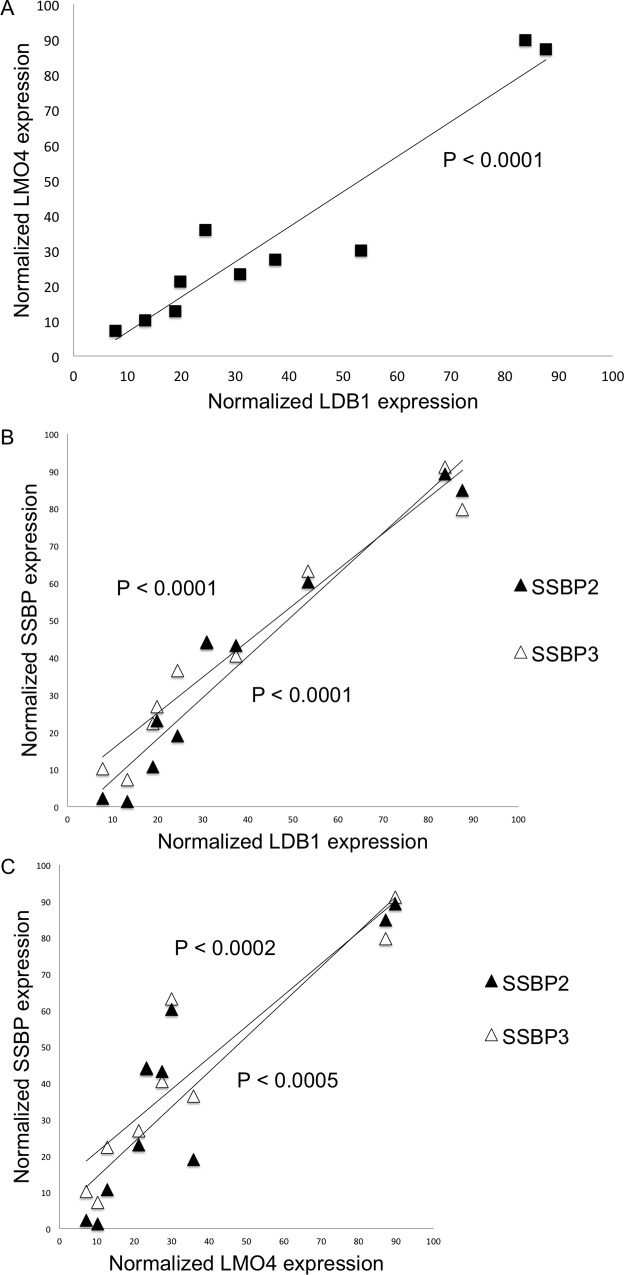
LMO4, LDB1, SSBP2, and SSBP3 abundance was highly correlated in a panel of human oral cavity carcinoma cell lines. The relative abundance of LMO4, LDB1, SSBP2, and SSBP3 in a panel of 10 human oral cavity carcinomas correlated significantly by immunoblot analysis. (A) LMO4 and LDB1 abundance were significantly correlated (r^2^ = 0.90, P < 0.0001, n = 10). (B) SSBP and LDB1 abundance were significantly correlated (black triangles, SSBP2, r^2^ = 0.95, P < 0.0001, n = 10; white triangles, SSBP3, r^2^ = 0.94, P < 0.0001, n = 10). (C) SSBP and LMO4 abundance were significantly correlated (black triangles, SSBP2, r^2^ = 0.80, P < 0.0005, n = 10; white triangles, SSBP3: r^2^ = 0.84, P < 0.0002, n = 10).

LMO4 and LDB1 abundance were shown by Mizunuma and colleagues to be increased and, of particular interest, concordant in human oral cavity carcinomas through semi-quantitative immunohistochemistry analysis [11]. This correlation in LMO4 and LDB1 expression, which would not be predicted or mandated by their physical interaction, prompted us to investigate whether these proteins were protected by SSBPs from turnover as we previously described [19]. After validating the specificity and establishing the optimum dilution of a LMO4 monoclonal antibody provided by Dr. Jane Visvader (Walter and Eliza Hall Institute, Victoria, Australia), expression of LMO4, LDB1, and SSBP was quantified with immunoblot analysis for 10 human oral cavity carcinoma cell lines. The names and sources of these lines are provided in Materials and Methods. Confirming published immunohistochemical studies [11], a highly significant correlation was observed between LMO4 and LDB1 protein abundance (r^2^ = 0.90, P < 0.0001) (Fig 1A). In addition, SSBP and LDB1 abundance (SSBP2 *vs*. LDB1, r^2^ = 0.95, P < 0.0001; SSBP3 *vs*. LDB1, r^2^ = 0.94, P < 0.0001) (Fig 1B) and SSBP and LMO4 abundance (SSBP2 *vs*. LMO4, r^2^ = 0.80, P < 0.0005; SSBP3 *vs*. LMO4, r^2^ = 0.84, P < 0.0002) were also significantly correlated (Fig 1C). Having established by overexpression and knock-down approaches that SSBPs inhibit poly-ubiquitylation of LDB1 and subsequent proteasomal destruction of LDB1 and LMO proteins in murine erythroid progenitors [19], these results suggest that SSBP2 and/or SSBP3 also control LDB1 and LMO4 accumulation in human head and neck cancer cells. Further, they define the range over which LMO4 and LDB1 levels are regulated in this tumor type and, lastly, provide proof of principle that a biological process discovered in the context of cellular differentiation also obtains in cancer [19].

Several of the lines in this panel, including SCC-4, SCC-9, and SCC-25 have been previously used in studies of metastasis and their ability to invade Matrigel characterized [30–32]. To assess, first, whether the abundance of these proteins correlated with invasiveness, a subset of our panel of human tumor cell lines were analyzed in the Matrigel chamber assay. Although knockout studies described below are more definitive, the invasive potential of these lines broadly correlated with LMO4, LDB1, and/or SSBP2/3 abundance (data not shown). Indeed, the two lines with the highest expression of these proteins, SCC-4 and VU-SCC-1729, were the most invasive in this assay, and one of these was selected for loss-of-function studies as described below.

### Expression of LMO4, LDB1, and SSBPs in human head and neck cancer cells metastatic to regional lymph nodes

**Fig 2:**
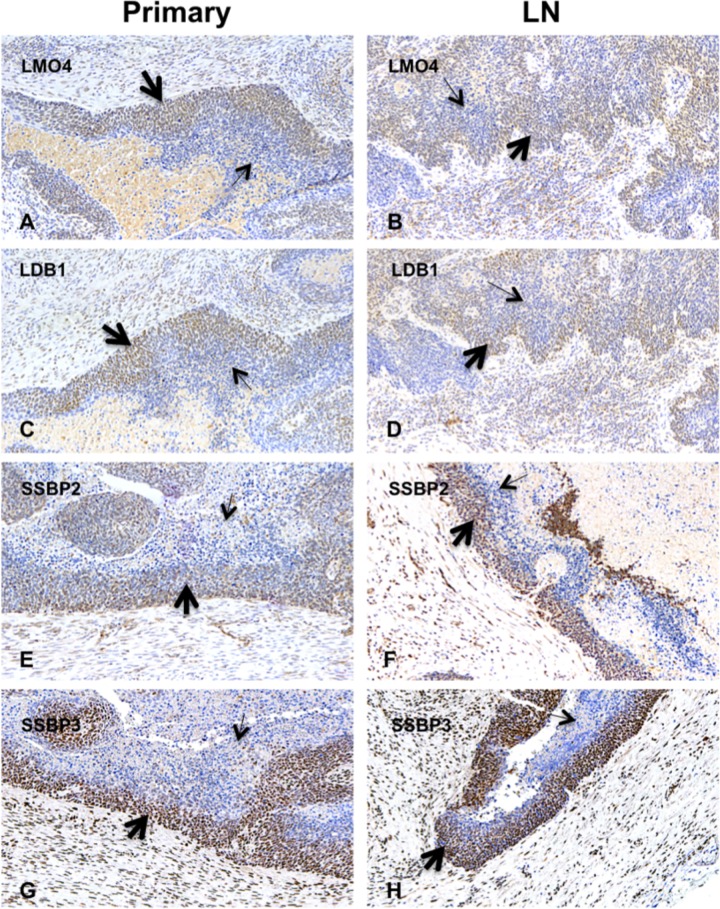
LMO4, LDB1, SSBP2, and SSBP3 expression was concordant in human oro-pharyngeal carcinomas and lymph nodes involved by metastasis. (A) LMO4, (C) LDB1, (E) SSBP2, and (G) SSBP3 expression was enriched at the invasive edge (thick arrows) of tumors compared to more central locations (thin arrows). (B) LMO4, (D) LDB1, (F) SSBP2, and (H) SSBP3 expression was detected in lymph nodes for all four carcinomas in which paired samples were available and exhibited a similar intracellular and intra-tumor distribution to the tumor primary. Control sections stained in parallel with secondary antibody alone or an isotype control antibody and secondary antibody were negative for histochemical reaction product (data not shown).

Based on immunolocalization in human oral cavity carcinomas, Mizunuma and colleagues proposed that LMO4 and LDB1 are involved in tumor metastasis. Specifically, they showed that LMO4 and LDB1 colocalized to nuclei of cells at the invasive front of oral cavity carcinomas and that LMO4 and LDB1 immunoreactivity was higher in cells metastatic to cervical lymph nodes than in cells from tumor primaries. To begin to investigate the role of SSBPs in the metastatic behavior of human head and neck cancer, SSBP2, SSBP3, LMO4, and LDB1 expression was compared in three pairs of oropharyngeal cancers and lymph nodes pathologically confirmed to be involved by cancer. Validating published results, nuclear LMO4 and LDB1 expression were detected by immunohistochemistry in every pair, and SSBP2 and SSBP3 expression were likewise concordant in tumor primaries (Fig 2A, 2C, 2E and 2G) and lymph nodes (2B, 2D, 2F and 2H). While these proteins appeared to co-localize in the same cells, the experiments were not designed to evaluate this point. In sum, these data precisely parallel the findings of Mizunuma and colleagues of concordant expression of LMO4 and LDB1 in human oral cavity carcinomas and lymph node metastases and extend theirs to include two other nuclear proteins, SSBP2 and SSBP3, and head and neck cancers from a second anatomical site. The latter finding is particularly notable since oral cavity and oropharyngeal carcinomas are differentially associated with HPV infection (see below).

### Reduced LMO4 protein abundance, cellular proliferation, and invasiveness in *LDB1* gene targeted oral cavity carcinoma cells

**Fig 3:**
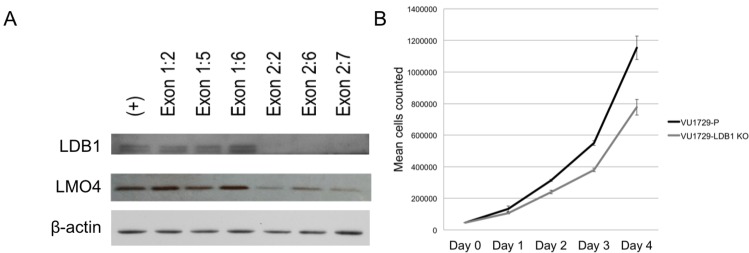
LDB1 and LMO4 protein abundance were reduced and cellular proliferation inhibited in LDB1 knockout cells. (A) LDB1 and, to a lesser extent, LMO4 protein abundance were reduced in VU-SCC-1729 cells in which *LDB1* was inactivated by CRISPR/Cas9 gene targeting (VU-SCC-1729 exon 2) but not in cells in which gene targeting was less effective (VU-SCC-1729 exon 1). (B) Cells in which the *LDB1* gene was inactivated (VU-SCC-1729-2:7) accumulated in significantly and progressively lower numbers relative to VU-SCC-1729 cells, starting on day 2 of culture (P = 0.053). +, vector control cells.

We next investigated the specific biological functions of LMO4 and LDB1 in head and neck cancer cells. Given problems with overexpression, with enforced expression of either LDB1 or LMO2 exerting dominant negative effects on erythroid differentiation [33, 34], we used a loss-of-function approach involving inactivation of the gene for LDB1. Because LDB1 can simultaneously contact both the LMO protein and SSBP [19], we reasoned that this would most effectively disrupt LMO4, LDB1, and SSBP-containing complexes (Fig 3A). LMO4 knockout, in contrast, would impact only LMO4 accumulation and SSBP knockout would impact LDB1 and, in particular, LMO4 abundance only indirectly. Further, since SSBP2 and SSBP3 appeared to have a role in stabilizing LDB1 in head and neck cancer cells, both SSBP genes would need to be targeted to maximally reduce the amount and function of this complex. Therefore, CRIPSR/Cas9-mediated gene conversion was used to disrupt exon 1 or exon 2 of the *LDB1* gene in VU-SCC-1729 cells. While LDB1 protein expression was partly reduced in exon 1-targeted cells, targeting of exon 2 resulted in complete loss of LDB1 expression (Fig 3A). This in turn was associated with significant reduction in LMO4 expression (Fig 3A), consistent with our previous studies using RNA interference [19]. A significant and progressive reduction in the number of knockout compared to control cells in culture was noted (Fig 3B), accompanied by a small, but not statistically significant, decrease in their viability (P = 0.053).

**Fig 4:**
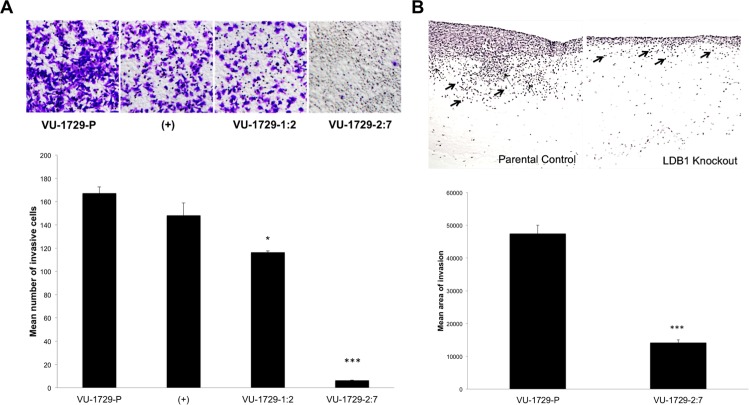
Loss of LDB1 expression in VU-SCC-1729 cells significantly reduced cellular invasiveness in two experimental models. (A) Invasion through reconstituted basement membrane (Matrigel) was evaluated for VU-SCC-1729 cells, vector control cells (+),VU-SCC-1729-1:2 cells in which LDB1 was only modestly knocked down, and VU-SCC-1729-2:7 cells in which LDB1 protein expression was almost completely ablated. (B) Invasion into a mixture of fibroblasts and extracellular matrix was evaluated in an organotypic reconstruct assay for parental and VU-SCC-1729-2:7 cells. The highly invasive VU-SCC-1729 cell line exhibited significant penetration of the fibroblast-collagen matrix by single cells (thick arrows) and cell clusters (thin arrows). The invasiveness of LDB1 knockout cells was significantly impaired, with only rare individual cells (thick arrows) and no clusters of cells penetrating the fibroblast layer. *, P < 0.01; ***, P < 0.0001.

LDB1-null VU-SCC-1729 cells were then evaluated in a gradient-dependent invasion assay. Compared to parental and vector control cells, LDB1 knockout cells were much less effective in invading and migrating through Matrigel under the conditions employed (P = 0.0011 and P = 0.0057, respectively). While the exon 1-targeted VU-1729-1:2 line was somewhat less invasive compared to the parental line (P = 0.0114), the exon 2-targeted line was profoundly impaired (Fig 4A), paralleling expression data. Thus, reduction in LDB1 and LMO4 protein abundance, and by inference an LDB1-, LMO4-, and SSBP-containing transcriptional complex, decreased Matrigel invasion *in vitro*.

To evaluate invasiveness in a more physiologic context, the parental and LDB1 knockout (VU-SCC-1729-2:7) lines were also tested in an organotypic reconstruct assay which permitted visualization, and enumeration, of oral cavity carcinoma cells capable of penetrating a suspension of esophageal fibroblasts, collagen, and basement membrane [26, 35]. For both lines there was evidence of individual tumor cells invading the fibroblast layer (Fig 4B, thick arrows), characteristic of epithelial-mesenchymal transition (EMT). In contrast, the number of cell clusters (Fig 4B, thin arrows) penetrating the fibroblast layer, characteristic of collective cell migration (or invasion), was significantly lower for the LDB1 knockdown compared to the parental line (P < 0.0001) (Fig 4B). Thus, data from two independent assays showed that depletion of LDB1 with resultant reduction in LMO4 expression significantly impaired invasive function.

### Reduced size and decreased vascularization of tumor xenografts of *LDB1* gene targeted human oral cavity carcinoma cells

**Fig 5:**
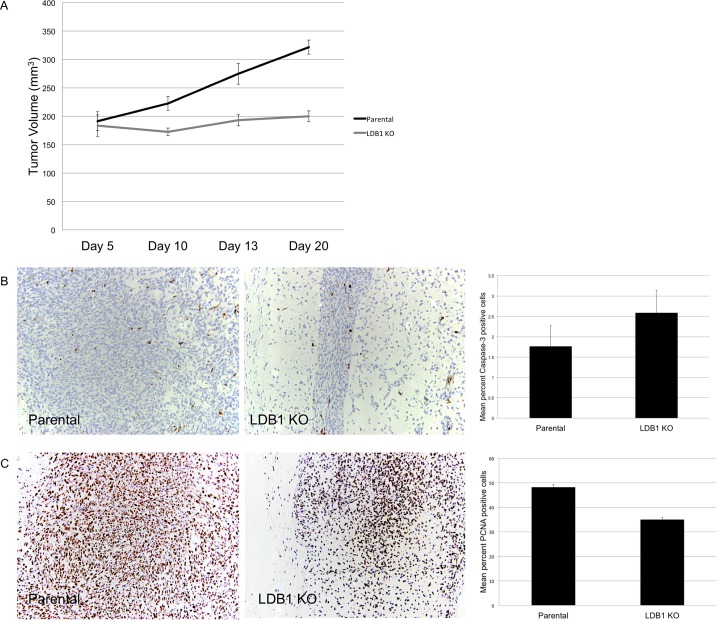
Loss of LDB1 expression in VU-SCC-1729 cells significantly reduced tumor growth in nude mice through an effect on cell proliferation. (A) Tumor growth in nude mouse xenografts was compared in VU-SCC-1729 cells and VU-SCC-1729-2:7 cells in which LDB1 protein expression was ablated. A difference in tumor size was first detected on day 10 (P = 0.002), which progressively increased from day 13 (P = 0.00096) to day 20 (P = 0.0000194) to day 23 (P = 0.0000345). (B) Caspase-3 staining for apoptosis was not significantly different between LDB1 KO and parental tumors (P = 0.2406). Aggregate data on caspase-3 staining are presented to the right of the image. (C) PCNA staining for proliferation was significantly reduced in LDB1 KO tumors compared to tumors derived from parental VU-SCC-1729 cells (P = 0.00028). Aggregate data on PCNA staining are presented to the right of the image.

To further evaluate the role of LIM-only and LIM domain interacting proteins in tumor cell function, limiting numbers of parental and LDB1 knockout (VU-SCC-1729-2:7) cells were injected into the flanks of nude mice, and tumor volumes were measured over the three weeks following. Although tumor size did not differ in the first 5 days, the volume of tumors derived from LDB1 knockout cells was significantly lower than in tumors from parental cells by day 10 (P = 0.002) and became progressively more divergent by day 13 (P = 0.00096), day 20 (P = 0.0000194), and day 23 (P = 0.0000345) (Fig 5A). Thus, LDB1 and LMO4 protein abundance appeared to be essential for tumor growth *in vivo*.

**Fig 6:**
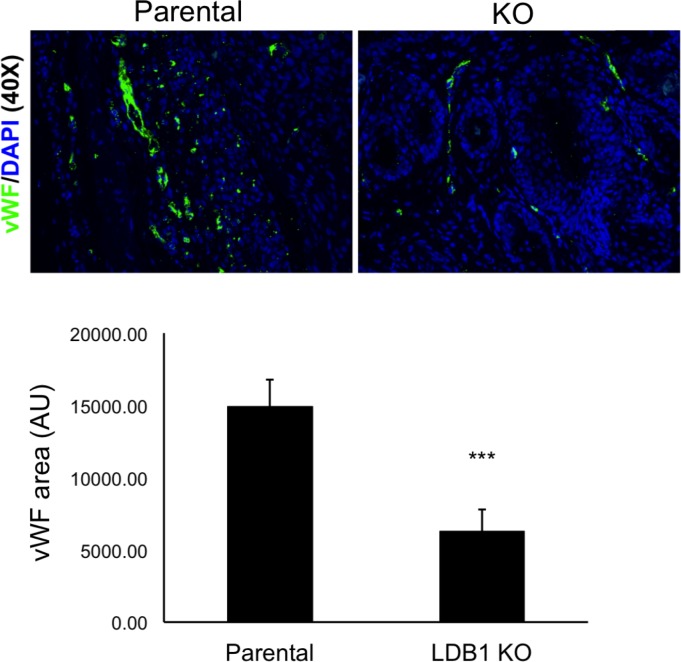
Loss of LDB1 expression in VU-SCC-1729 cells significantly reduced angiogenesis in tumor xenografts in nude mice. Endothelial cell numbers were compared in nude mouse xenografts from VU-SCC-1729 cells and VU-1729-2:7 cells in which LDB1 protein expression was eliminated (LDB1 KO). Endothelial cells were stained with antibody to vWF (green immunofluorescence) and nuclei counterstained with DAPI (blue immunofluorescence). The number of vWF-expressing endothelial cells was significantly reduced in LDB1 KO compared to control tumors (P = 0.00196).

Next, to determine whether these differences in tumor size resulted from altered apoptosis and/or proliferation, tumors were harvested on day 23, sectioned, and stained with antibodies to caspase-3 and PCNA, respectively. While tumors derived from LDB1 knockout cells showed a slight increase in the frequency of cells stained with the apoptosis marker caspase 3, this difference was not statistically significant (P = 0.2406) (Fig 5B). In contrast, tumors from knockout cells exhibited significantly less staining for the proliferation marker PCNA (Fig 6C), paralleling the reduced proliferation of LDB1-deficient VU-SCC-1729 cells *in vitro* (Fig 3B).

In addition to being smaller in size, tumors derived from LDB1 knockout cells appeared to be less vascularized (not shown), and LDB1-null tumors showed significantly less staining with the endothelial marker vWF compared to parental tumors (P = 0.00196) (Fig 6). Altogether, these data indicate that loss of LDB1 decreased both proliferation and vascularization in tumor xenografts of a human head and neck cancer cell line.

**Fig 7:**
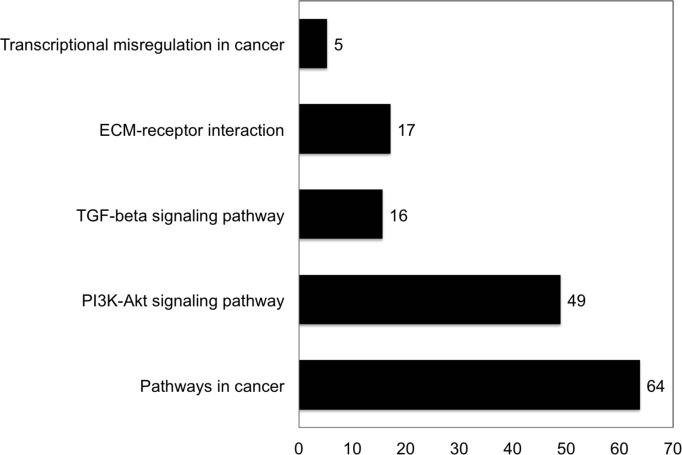
Biological processes highlighted by iPathway Guide from differentially expressed genes in LDB1 knockout compared to wild-type VU-SCC-1729 cells. Numbers represent percentage of genes in each category differentially expressed in KO *vs*. wild-type cells using a threshold of 0.05 for statistical significance and 2 for absolute log expression change. P values for the pathways identified were: ECM-receptor interaction (P = 0.0000527), pathways in cancer (P = 0.001), TGF-β signaling (P = 0.002), transcriptional misregulation in cancer (P = 0.002), and PI3K-Akt signaling (P = 0.003).

Finally, RNA-seq analysis was carried out to identify the RNAs altered by loss and reduction, respectively, in LDB1 and LMO4 expression and differentially expressed RNAs grouped according to the cellular processes to which they were predicted to contribute. We found, first, that certain RNAs were upregulated and others down-regulated, in accord with LMO2 and LDB1-containing complexes both activating and repressing transcription [36–38]. Second, several processes critical for tumor promotion and spread, including angiogenesis (data not shown), matrix-cell interactions, and TGF-β and phosphatidylinositol-3-kinase (PI3K) signaling (Fig 7), were highlighted. Indeed, PI3K (reviewed in [39]) and TGF-β (reviewed in [40]) signaling have been previously recognized to promote head and neck cancer cell proliferation and survival and also tumor invasion, metastasis, and angiogenesis, most likely in series [41]. Since LMO4, LDB1, and the SSBPs contribute to a common transcriptional complex(es), some of these genes likely represent direct targets of these complexes.

## Discussion

The ability of cancer cells to invade and migrate into normal tissue is essential to both local spread and metastasis. Acquisition of these functions requires transcriptional reprogramming, with repression of genes important for epithelial identity and activation of those promoting mesenchymal function. Indeed, similar gene expression patterns have been observed in association with two processes thought to drive metastasis, epithelial-mesenchymal transition [42] and collective cell migration (or invasion) [43]. In the present study, we confirm that the the abundance of LIM-only protein LMO4 and LIM domain-binding protein LDB1 are frequently increased in carcinoma of the head and neck and suggest that their aberrant expression is mediated at a post-translational level by two single-stranded DNA-binding proteins, SSBP2 and SSBP3. Finally, we demonstrate the involvement of LMO4 and LDB1 in cancer cell proliferation, invasiveness, migration, and angiogenesis, suggesting these nuclear proteins are important regulators of growth and metastasis in HNSCC.

While these studies add to a considerable literature demonstrating that LMO and associated LIM domain binding proteins can be pro-oncogenic [5, 13, 16, 44], less is known about the involvement or function of SSBPs in cancer. Although increased SSBP2 expression was associated with shorter survival in glioblastoma [45], SSBP2 was also shown to inhibit the growth of prostate cancer cells [46] and to act as a tumor suppressor in myeloid leukemias [21]. Depending on the context, then, the SSBPs could be oncoproteins or tumor suppressors.

Carcinoma of the oropharynx is more frequently associated with HPV infection and less frequently associated with tobacco use compared to oral cavity carcinoma [47–50], with viral infection associated with specific genetic alterations and increased responsiveness to radiation and chemotherapy. The similarity in the frequency and localization of LMO4, LDB1, and SSBP2/3 expression in oral cavity and oropharyngeal carcinomas, by contrast, suggest these proteins subserve common functions in HNSCC regardless of etiology.

LMO2 [51], LDB1 [52], and SSBP2 [53] are required for the function or maintenance of hematopoietic stem cells, and Ldb1 has also been shown to be essential to the survival of intestinal epithelial stem cells [54]. As the malignant counterparts of these cells have been found in a number of epithelial neoplasms, including HNSCC (reviewed in [55]) and have been implicated in metastasis [56, 57], tumor angiogenesis [58], and lymphangiogenesis [59] (see below), we speculate that LIM-only, LIM domain-binding, and single-stranded DNA-binding proteins regulate both normal and cancer stem cells.

A role for LDB1 [11] and its LIM domain partner protein LMO4 [10, 11] in metastasis was suggested from the location of these proteins in oral cavity carcinoma, a finding which we extended to also include oropharyngeal carcinoma. A function in tumor angiogenesis had not been previously described, however, and while LMO2 has been implicated in physiologic [60] and pathologic [44, 61, 62] angiogenesis, these studies provide the first evidence for LMO4 involvement in this process. This may have relevance to other epithelial malignancies in which this LIM-only protein is overexpressed, in particular carcinoma of the breast.

SSBP2, SSBP3, and SSBP4 are have been suggested to bind stretches of single-stranded DNA, and the gene encoding the first mammalian SSBP discovered was cloned as a result of this property [63]. However, single-stranded DNA-binding activity proved dispensable for its LDB1 and LMO protein stabilizing function [19], and SSBP2 and SSBP3 can be recruited to DNA by transcription factors, including basic helix-loop-helix proteins in erythroid cells [19] and LIM-homeodomain proteins in pituitary cells [64, 65]. An important issue is the identity of the transcription factor(s) that recruit these adapter proteins to DNA in cancer cells and the genes regulated by these complexes. We are utilizing chromatin immunoprecipitation in conjunction with exonuclease digestion and next-generation sequencing analysis to elucidate the specific DNA sequences bound by these complexes, which we hope to leverage toward identification of the proteins that recruit them to DNA. Greater understanding of the mechanisms by which LMO4, LDB1, and SSBPs regulate tumor cell biology will be important for developing new treatments against this invasive epithelial malignancy.
